# Upper Tract Urothelial Carcinomas in Patients with Chronic Kidney Disease: Relationship with Diagnostic Challenge

**DOI:** 10.1155/2014/989458

**Published:** 2014-08-07

**Authors:** Li-Jen Wang, Shen-Yang Lee, Bin Tean Teh, Cheng-Keng Chuang, Joëlle Nortier

**Affiliations:** ^1^Department of Medical Imaging and Intervention, Linkou Chang Gung Memorial Hospital, 5 Fu-Hsing Street, Gueishan, Taoyuan 33333, Taiwan; ^2^Department of Medical Imaging and Radiological Sciences, College of Medicine, Chang Gung University, Taoyuan 33333, Taiwan; ^3^Institute of Radiological Research, College of Medicine, Chang Gung University, Taoyuan 33333, Taiwan; ^4^Department of Nephrology, Linkou Chang Gung Memorial Hospital, Taoyuan 33333, Taiwan; ^5^Department of Medicine, College of Medicine, Chang Gung University, Taoyuan 33333, Taiwan; ^6^National Cancer Center and Duke-NUS Graduate Medical School, Singapore 169610; ^7^Department of Urology of Linkou Chang Gung Memorial Hospital, Taoyuan 33333, Taiwan; ^8^Department of Nephrology, Erasme Hospital, 1070 Brussels, Belgium

## Abstract

Chronic kidney disease and upper tract urothelial carcinomas display a bidirectional relationship. Review of the literature indicates that early diagnosis and correct localization of upper tract urothelial carcinomas in dialysis patients and kidney transplant recipients are important but problematic. Urine cytology and cystoscopy have limited sensitivity for the diagnosis of upper tract urothelial carcinomas in dialysis patients. Enhanced computed tomography and magnetic resonance imaging could prove useful for the detection and staging of upper tract urothelial carcinomas in dialysis patients. Renal ultrasound can detect hydronephrosis caused by upper tract urothelial carcinomas in kidney transplant recipients but cannot visualize the carcinomas themselves. High detection rates for upper tract urothelial carcinomas in kidney transplant recipients have recently been demonstrated using computed tomography urography, which appears to be a promising tool. To detect carcinomas in dialysis patients and kidney transplant recipients as early as possible, regular screening in asymptomatic patients and diagnostic work-up in symptomatic patients should be performed using a combination of urological and imaging methods. Careful assessment of subsequent recurrence within the contralateral upper urinary tract and the urinary bladder is necessary for dialysis patients and kidney transplant recipients with upper tract urothelial carcinomas.

## 1. Introduction

Chronic kidney disease (CKD) is characterized by the presence of kidney damage or decreased kidney function with a glomerular filtration rate of <60 mL/min/1.73 m^2^ for at least 3 months, irrespective of the cause [1]. The definition of CKD underwent minor modifications in 2004 and now includes classification based on dialysis treatment or transplantation to denote the special care required for these groups of patients [2].

Upper tract urothelial carcinomas (UTUCs) and CKD are closely associated. High prevalence rates of CKD of 58.6% and 57.7% in China and Taiwan, respectively, have been reported in patients with UTUCs [3, 4]. The high prevalence of CKD in upper tract urothelial carcinoma (UTUC) patients is attributable to old age, aristolochic acid nephropathy, and increased risk status after nephroureterectomy, a standard treatment for UTUCs [5]. Similarly, Hung et al. demonstrated a linear relationship between UTUC prevalence and CKD severity. The prevalence rates of UTUCs in none/mild CKD, stage 3 CKD, and stage 4/5 CKD are 11%, 55%, and 71%, respectively, and these rates are significantly different from each other (*P* < 0.001) [6]. The bidirectional relationship between CKD and UTUCs suggests they may share risk factors.

Several nephrotoxins, including analgesics and aristolochic acid, might explain the bidirectional relationship between CKD and UTUCs in accordance with their nephrotoxic and carcinogenic effects [7]. Abuse of compound analgesic agents (mainly containing phenacetin) is associated with analgesic nephropathy and UTUCs in kidney transplant recipients (KTRs). Analgesic nephropathy is characterized by chronic renal interstitial nephritis with resultant renal functional impairment (in approximately 80% of cases) or even progression to end-stage renal disease (in approximately 10% of cases). KTRs with analgesic nephropathy have an increased risk of urothelial carcinomas (UCs), characterized by progressive upper urinary tract involvement [8]. UCs are more prevalent in female KTRs [8]. The proportion (2.8%, 7/250) of UCs in KTRs with analgesic nephropathy is significantly higher than that (0.49%, 7/1424) in KTRs without analgesic nephropathy [8]. In that study, all seven KTRs with UCs and analgesic nephropathy were female; moreover, they all experienced subsequent UTUCs following initial bladder involvement. In contrast, all seven KTRs with UCs and nonanalgesic nephropathy were male and, furthermore, they only had bladder UCs [8]. However, following the commercial withdrawal of compound analgesics containing phenacetin, a trend toward a decreased incidence of renal pelvic UCs has been observed [7, 9].

Aristolochic acid is a powerful nephrotoxin and human carcinogen, which also explains the association between CKD and UTUCs [10, 11]. Aristolochic acid nephropathy, first reported in Belgium, is characterized by chronic tubulointerstitial nephritis (in 93.3% of cases), which may progress to end-stage renal disease [12–14]. A variety of Chinese herbal remedies containing aristolochic acid for weight loss and a plethora of other ailments, including menstrual symptoms, snakebites, rheumatism, arthritis, and gout (especially in females), have been proven popular in Taiwan and China, among other countries [10, 11, 14]. A recent study in 152 Taiwanese UTUC patients revealed high prevalence rates for aristolactam deoxyribonucleic acid (DNA) adducts and p53 mutations, which serve as biomarkers of aristolochic acid exposure and demonstrate a close association between aristolochic acid exposure and UTUCs [10]. Recently, Balkan endemic nephropathy has been categorized as a form of aristolochic acid nephropathy due to the identification of aristolochic acid in* Aristolochia clematitis* [12, 13, 15]. Balkan endemic nephropathy is associated with a 100-fold increased frequency of UTUCs compared with nonendemic areas [16]. Consumption of contaminated or aristolochic acid containing Chinese herbal remedies or foods raises the likelihood of developing UTUCs and CKD; accordingly, banning its use has become an important global public health issue.

## 2. Upper Tract Urothelial Carcinomas in Dialysis Patients: A Diagnostic Challenge

Urothelial carcinoma (UC) is the most frequently presenting malignancy in dialysis patients in Taiwan and Balkan endemic nephropathy areas [17]. Dialysis patients also have a higher cancer risk. In dialysis and end-stage renal disease patients, DNA repair impairment, immune dysfunction, and antioxidant defense reduction, in addition to carcinogen accumulation and chronic infection or inflammation, are all potential factors for increased cancer risk [18]. Overall cancer risk in dialysis patients is 10–80% higher compared with the general population [19, 20]. The prevalence of each type of cancer in dialysis patients varies according to geographical area. Overall, renal cell carcinoma represents the most common urinary tumor in dialysis patients in Europe, Australia, and New Zealand [21]. In contrast, UCs present with unusual frequency (0.9–1.7%) in dialysis patients in Taiwan and Balkan endemic nephropathy areas, compared with patients in Europe, Australia, and New Zealand (0.3–0.6%, Table 1) [17, 21–25]. UCs in dialysis patients in Taiwan and Balkan endemic nephropathy areas are observed predominantly in females (53.8–70.6%; median, 61.3%) and upper tract (31.6–62.5%; median, 52.9%, Table 1). They are associated with both consumption of aristolochic acid in females and Balkan endemic nephropathy [17, 21–25].

Diagnosis of UTUCs in dialysis patients represents a clinical challenge, especially in the early stages. The most commonly presenting symptom is gross hematuria, that is, “bloody urethral discharge,” observed in over 90% of patients [26, 27]. However, heparinization for dialysis also results in hematuria [25]. Hematuria in dialysis patients signals the need of further investigation but does not lead to a specific diagnosis. Invasive UTUCs in dialysis patients are associated with a reduced likelihood of survival [26, 27]. Therefore, correct and early diagnosis of UTUCs represents an important goal.

Reported detection rates for UCs and UTUCs in dialysis patients have varied markedly across a number of urological and imaging studies (Table 2) [17, 23, 28]. Furthermore, these results provide information pertaining only to true-positive (detection rates) and false-negative rates [17, 23, 28]. Traditionally, urine cytology, retrograde pyelography, and cystoscopy have been used to diagnose UCs in dialysis patients [26]. Urine cytology is associated with an extremely low detection rate (0.0–33.3%) for UCs, especially UTUCs, in dialysis patients [17, 23, 28]. It is also impractical to use urine cytology to screen for UCs and UTUCs, because the majority of dialysis patients experience anuria [23]. Furthermore, although urine cytology can indicate the presence of UC, it cannot differentiate UTUCs from bladder UCs. Diagnosis of UTUCs during retrograde pyelography is primarily based on filling defects in the upper urinary tracts, with imaging characteristics suggestive of UTUCs. The detection rates for UCs and UTUCs in dialysis patients using retrograde pyelography have been reported as 75.0% and 85.7%, respectively [17, 23]. Detection rates for retrograde pyelography may be overestimated due to the exclusion of cases involving technical failure or insufficient diagnostic information. Technical failure usually refers to the inability to identify or catheterize ureteral lumen using cystoscopy, due to ureter atrophy or ureter trauma by ureter fibrosis [29, 30]. Tumors or blood located in more distal parts of the ureter can hinder the detection of proximally located UTUCs, leading to insufficient diagnostic information pertaining to retrograde pyelography [29, 30]. Cystoscopy has principally been used to detect bladder UCs rather than UTUCs. The majority of UTUCs are not detectable using cystoscopy, with the exception of ureteral UCs protruding into the bladder cavity via the ureteral orifice. Therefore, retrograde pyelography might be a more reliable tool for detecting UTUCs compared with urine cytology and cystoscopy. A combination of cystoscopy and retrograde pyelography might be capable of detecting the majority of bladder UCs and UTUCs in dialysis patients.

Recently, computed tomography (CT) and magnetic resonance imaging (MRI) have been proposed for use in the diagnosis and staging of UTUCs in dialysis patients [26]. The detection rates for UCs using CT and MRI have recently been reported as 82.4% and 94.9%, respectively (Table 2) [17]. For dialysis patients experiencing anuria, contrast-enhanced CT can be performed without the concern of contrast-induced nephropathy, because the renal functions of these patients are particularly compromised. The contrast-enhancing nature of UTUCs renders them distinguishable from nonenhancing hematoma; moreover, their presence on contrast-enhanced CT is highlighted by amplified differences in density between UTUCs and surrounding normal tissue. Using unenhanced MRI, UTUCs in dialysis patients exhibit variable signal intensities, which help distinguish them from adjacent, normal structures. Similar to contrast-enhanced CT, the use of a gadolinium-based contrast agent for enhanced MRI in dialysis patients would also help in the detection of UTUCs. However, three gadolinium-based contrast agents are associated with the greatest number of nephrogenic systemic fibrosis cases; thus, their application is contraindicated in dialysis patients [31]. Instead, other gadolinium-based contrast agents should be employed if there is a strong clinical need for enhanced MRI. In summary, CT and MRI could be valuable tools for detecting UTUCs in dialysis patients. However, large-scale studies focusing on the diagnostic accuracy of CT and MRI are necessary to corroborate their efficacy.

Detection of recurrent UCs in dialysis patients with initial UTUCs during follow-up is very important if prophylactic total urinary tract extirpation has not been performed [27]. Recurrence of contralateral UTUCs and bladder UCs in dialysis patients with UTUCs is very common, occurring in 31.1–37.9% and 52.6% of patients, respectively [26, 27, 32]. To completely eliminate the need to detect recurrence, Wu et al. suggested the performance of total urinary tract extirpation during one-stage surgery in the treatment of dialysis patients with UTUCs. However, higher perioperative mortality rates are observed in dialysis patients with cystectomy, compared with dialysis patients without cystectomy [27]. For dialysis patients with UTUCs and preserved urinary bladders, a regular cystoscopy, every 3 months for the first 2 years and annually thereafter, has been suggested for the detection of recurrence in the urinary bladder [27]. For dialysis patients with initially unilateral UTUCs who have undergone unilateral nephroureterectomy only, enhanced CT or MRI might represent a useful, noninvasive means of detecting recurrence in the contralateral upper urinary tract.

## 3. Upper Tract Urothelial Carcinomas in Kidney Transplant Recipients: A Diagnostic Challenge

The incidence of UCs in KTRs varies by geographic area. In China and Taiwan, the proportion of UCs in KTRs is unusually high: 0.9–4.1% versus 0.1–1.1% in other regions (Table 3) [33–51]. Impaired immunity due to immunosuppressants, viral infection, and uremia has been proposed as a risk factor for the development of malignancies in KTRs [35], which have a malignancy incidence rate approximately 4-5-fold higher than that for the general population [35, 50, 52]. In the majority of areas, the most frequent malignant tumors in KTRs are lymphomas, followed by skin cancer [35]. However, in KTRs in Taiwan and China, UCs are the most common presenting malignancy, predominantly in females (58.3–81.0%; median, 70.6%) [33–42]. A high proportion (40.7–93.3%; median, 80.7%) of native UTUCs and the involvement of multifocal sites (i.e., ≧two organs of the urinary tract) are also observed (Table 3) [33–42]. Early recurrence in the urinary bladder or contralateral upper urinary tract is also common in UCs in KTRs in China and Taiwan [33–42]. These features of UCs in KTRs in China and Taiwan are quite different from those of the predominant bladder UCs and from the male predominance of UCs observed for KTRs in other areas [33–51].

The prognosis of KTRs with UTUCs depends primarily on the tumor stage and tumor grade [37, 38]. However, it is difficult to diagnose UCs in KTRs at an early stage, especially UTUCs of the native upper urinary tract, according to clinical symptoms alone [35]. Invasive UTUCs are often characterized by progressive multifocal recurrence, even following adjunctive radiotherapy or systemic chemotherapy, and patients may ultimately die due to metastasis [46, 47]. In contrast, noninvasive UTUCs are associated with low recurrence and mortality rates [46, 47]. UCs in KTRs present with a wide variety of symtoms or are sometimes asymptomatic [37–40, 51, 53]. Painless gross hematuria represents the most common symptom, followed by microscopic hematuria and chronic urinary tract infection [37–40, 51, 53]. Occasionally, KTRs with UCs present with fever, urinary retention, weight loss, or bone pain [51, 53]. Unfortunately, these clinical symptoms are nonspecific to UCs and UTUCs [37–40, 51, 53]. Other urinary diseases and even healthy KTRs can also present with these symptoms [37–40, 51, 53]. Furthermore, asymptomatic KTRs with UCs account for 11.4–45.5% of cases [37, 51, 53]. Therefore, diagnosis of UTUCs in KTRs according to the clinical symptoms often leads to delayed diagnoses [35–37, 40].

A variety of urological and imaging methods have been employed traditionally in the diagnosis of UCs and UTUCs in KTRs, but they are all characterized by certain shortcomings [35–37, 40, 51, 53]. The detection rates for UCs and UTUCs in KTRs using these methods are provided in Table 4 [35–37, 40, 51, 53]. Urine cytology collected from spontaneously voided urine is associated with a wide range of detection rates (8.3–81.5%; median, 47%) for UCs in KTRs [35–37, 40, 51, 53]. The reported low detection rate for UCs in KTRs using urine cytology could be explained by poor functioning of the native upper urinary tracts [35, 40, 53]. Renal ultrasound of native kidneys usually reveals secondary hydronephrosis rather than UTUCs themselves, which is particularly facilitative for the diagnosis of UTUCs in asymptomatic patients [36, 37, 40, 51, 53]. Renal ultrasound alone, however, detected only 9.1–53.6% of UTUCs in KTRs [36, 37, 40, 51, 53]; accordingly, it usually serves as a complementary tool only in the detection of native UTUCs. Cystoscopic and ureteroscopic biopsy detected 38% and 50% of UCs in KTRs, respectively [35]. However, ureteroscopy confers a risk of native upper urinary tract rupture due to inherent poor elasticity [53]. The invasive nature of cystoscopy and ureteroscopy renders them unsuitable routine screening tools for UCs and UTUCs in KTRs; they are typically used to confirm the presence of UCs in symptomatic KTRs [29, 40]. In summary, the use of a single traditional method alone is likely to underestimate UC and UTUC occurrence in KTRs. Urine cytology combined with abdominal ultrasound has been used to screen for UCs in KTRs but detected only one-third of UCs; moreover, these UCs were advanced, and the mortality rate was commensurately high [53]. In contrast, a detection rate of 96.7%, for UCs in KTRs using a combination of cystoscopy and retrograde pyelography, has been reported [40]. This approach is justified in clinically suspected patients, but its feasibility for the screening of UCs in KTRs remains contentious due to its invasiveness.

**Figure 1:**
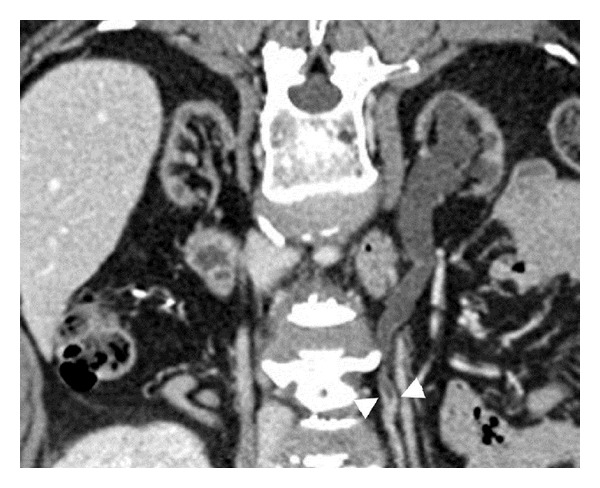
Curved planar reformatted image of computed tomography urography of a 56-year-old female kidney transplant recipient exhibits a fork sign (arrowheads) in the left native ureter representing a left ureteral UC.

**Figure 2:**
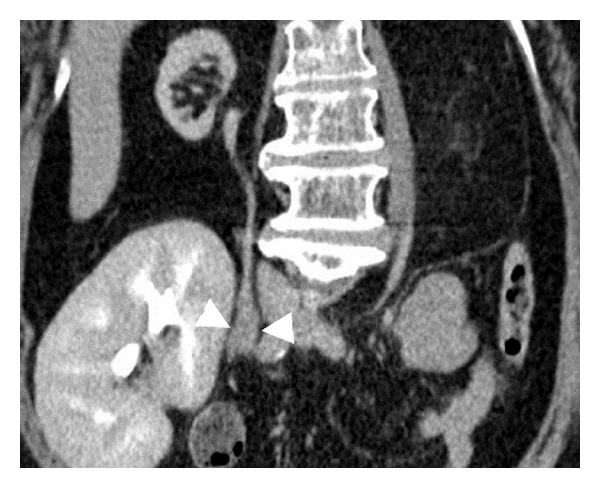
Curved planar reformatted image of computed tomography urography of a 55-year-old female kidney transplant recipient exhibits a spindle sign in the right native ureter (arrowheads) indicative of urothelial carcinoma.

With advancements in imaging technology, CT urography is now a promising tool for the detection of UTUCs in KTRs. CT urography has high specificity (93–99%) and moderate-to-high sensitivity (67–100%) in hematuria patients with sufficient renal function [30, 54–61]. CT urography is also more accurate than excretory urography for diagnosing UTUCs in these patients [56, 61]. Therefore, CT urography has been recommended as a first-line imaging tool for diagnosing UTUCs, due to its high accuracy and noninvasive nature [58, 62]. Does CT urography display similar results for the detection of UTUCs in KTRs? The detection rate has been reported as 85.7% (Table 4) [63], lower than that (95.8%) seen in hematuria patients (non-KTRs) [61, 63] but nonetheless probably higher than those associated with other more traditional methods. Smaller UTUC sizes and poor contrast opacification of native upper urinary tract in KTRs may explain the lower detection rate of CT urography in KTRs. Two novel indicators of native ureteral UCs in KTRs have been identified using CT urography [63]; the fork sign (Figure 1) indicates ureteral UCs with proximal dilatation [64]. Conversely, the spindle sign (Figure 2) is indicative of ureteral UCs in nondilated native ureters, which deform the corresponding ureteral segment into spindle shapes [64]. Additional CT urography studies employing large numbers of KTRs are necessary to corroborate its efficacy in the detection of UTUCs.

A pretransplantation survey of the urinary tract using cystoscopy, CT urography, renal ultrasound, and urine cytology should be conducted to exclude the presence of UC in renal transplantation candidates. The shortest interval between subsequent UC diagnosis following renal transplantation has been reported to be 2 months, suggesting that these UCs probably existed prior to renal transplantation [35, 37, 38]. KTRs with preexisting UTUCs may have a poor prognosis, with early tumor dissemination to lymph nodes and distant organs despite aggressive treatment using bilateral nephroureterectomy [37]. Therefore, by undergoing a standard pretransplantation urological and imaging survey, the risk of preexisting UCs in KTRs could be reduced. Furthermore, the results of standard pretransplantation surveys could serve as a baseline to highlight any interval changes following renal transplantation.

## 4. Conclusion

CKD and UTUC share a bidirectional association. Analgesics and aristolochic acid are common risk factors for CKD and UTUCs due to their nephrotoxic and carcinogenic effects. The ban on aristolochic acid containing medicines and foods to decrease aristolochic acid nephropathy and associated UTUCs has become an important global public health issue. Early and correct diagnosis of UTUCs in dialysis patients and KTRs is important but difficult. Urine cytology and cystoscopy have limited sensitivity for the diagnosis of UTUCs in dialysis patients. Enhanced CT and MRI could prove useful for the detection and staging of UTUCs in dialysis patients. Renal ultrasound is useful to detect hydronephrosis in KTRs with asymptomatic UTUCs; however, hydronephrosis is not specific to UTUCs. CT urography represents a promising tool to detect UTUCs in KTRs due to its high detection rate. Prior to renal transplantation, a urinary tract survey should be performed for the early detection of preexisting UTUCs. Regular screening of asymptomatic patients and diagnostic work-up for symptomatic patients using a combination of urological and imaging methods should be performed to achieve early diagnosis of UTUCs in dialysis patients and KTRs. Careful assessment of subsequent recurrence within the contralateral upper tract and the urinary bladder is essential in dialysis patients and KTRs with UTUCs.

## Figures and Tables

**Table 1:** Proportions, locations, and gender distribution of urothelial carcinomas in dialysis patients.

Reference (country)	Proportions in dialysis patients			Proportions in urothelial carcinoma patients	
	Dialysis patients (*n*)	Urothelial carcinoma (%)	Upper tract urothelial carcinoma (%)	Female urothelial carcinoma (%)	Upper tract urothelial carcinoma (%)
Stewart et al. (Europe) [21]	296903	825 (0.3%)	165 (0.1%)	NA	165 (20.0%)
Stewart et al. (Australia and New Zealand) [21]	13497	87 (0.6%)	34 (0.3%)	NA	34 (39.1%)
Wang et al. (Taiwan) [17]	10890	98 (0.9%)	31 (0.3%)	65 (66.3%)	31 (31.6%)
Ou et al. (Taiwan) [25]	1910	17 (0.9%)	9 (0.5%)	12 (70.6%)	9 (52.9%)
Chen et al. (Taiwan) [23]	1333	16 (1.2%)	7 (0.5%)	9 (56.3%)	7 (43.8%)
Chang et al. (Taiwan) [22]	1537	26 (1.7%)	14 (0.9%)	14 (53.8%)	14 (53.9%)
Cuckovic et al. (Serbia) [24]	923	16 (1.7%)	10 (1.1%)	NA	10 (62.5%)

NA: not available.

**Table 2:** Detection rates for urothelial carcinomas and upper tract urothelial carcinomas in dialysis patients using urological and imaging methods.

Patients	Test	Positive result (*n*)/total number (*N*)	Detection rate (%)	Reference
Urothelial carcinoma	Urine cytology	7/24	29.2%	Wang et al. [17]
		2/6	33.3%	Chen et al. [23]
	Retrograde pyelography	21/28	75.0%	Wang et al. [17]
	Cystoscopy	67/80	80.7%	Wang et al. [17]
	Computed tomography	42/51	82.4%	Wang et al. [17]
	Magnetic resonance imaging	37/39	94.9%	Wang et al. [17]
	Cystoscopy and/or retrograde pyelography	14/16	87.5%	Chen et al. [23]
	Computed tomography and endoscopy	16/16	100.0%	Satoh et al. [28]

Upper tract urothelial carcinoma	Urine cytology	0/10	0.0%	Satoh et al. [28]
	Retrograde pyelography	6/7	85.7%	Chen et al. [23]
	Computed tomography and endoscopy	10/10	100.0%	Satoh et al. [28]

*n*: number of patients with positive results in each diagnostic test.

*N*: number of patients who underwent each diagnostic test.

**Table 3:** Proportions, locations, and gender distribution of UCs and UTUCs in kidney transplant recipients.

Reference (country)	Proportion in kidney transplant recipients		Proportion in kidney transplant recipients with urothelial carcinomas		
	Kidney transplant recipients (*n*)	Urothelial carcinoma (%)	Female (%)	Multifocal (%)	Upper tract urothelial carcinoma (%)
Einollahi et al. [44] (Iran)	5532	7 (0.1%)	2 (28.6%)	0 (0.0%)	0 (0.0%)
Hoshida et al. [47] (Japan)	1744	2 (0.1%)	0 (0.0%)	0 (0.0%)	0 (0.0%)
Cox and Colli [43] (USA)	5920	11 (0.2%)	2 (18.2%)	0 (0.0%)	1 (9.1%)
Elkentaoui et al. [45] (France)	1350	5 (0.4%)	0 (0.0%)	0 (0.0%)	0 (0.0%)
Karczewski et al. [48] (Poland)	836	3 (0.4%)	1 (33.3%)	0 (0.0%)	0 (0.0%)
Rogers et al. [50] (UK)	1647	8 (0.5%)	NA	0 (0.0%)	0 (0.0%)
Gaya et al. [46] (UK)	274	3 (1.1%)	NA	0 (0.0%)	0 (0.0%)
Melchior et al. [49] (Germany)	802	8 (1.0%)	NA	0 (0.0%)	2 (25.0%)
Tsaur et al. [51] (Germany)	2001	21 (1.1%)	12 (57.1%)	4 (19.1%)	6 (28.6%)
Liu et al. [38] (China)	2572	24 (0.9%)	14 (58.3%)	15 (62.5%)	21 (87.5%)
Hao et al. [33] (China)	1945	19 (1.0%)	NA	7 (36.8%)	15 (79.0%)
Hu et al. [34] (China)	1293	21 (1.6%)	17 (81.0%)	9 (42.9%)	15 (71.4%)
Li et al. [36] (China)	1429	27 (1.9%)	21 (77.8%)	3 (11.1%)	11 (40.7%)
Xiao et al. [41] (China)	3790	100 (2.6%)	NA	53 (58.9%)∗	68 (75.6%)
Liao et al. [37] (Taiwan)	663	17 (2.6%)	NA	11 (64.7%)	14 (82.4%)
Wang et al. [39] (Taiwan)	320	10 (3.1%)	8 (80.0%)	4 (40.0%)	6 (60.0%)
Zhang et al. [42] (China)	3462	112 (3.2%)	NA	69 (61.6%)	93 (83.0%)
Kao et al. [35] (Taiwan)	670	24 (3.6%)	15 (62.5%)	19 (79.2%)	21 (87.5%)
Wu et al. [40] (Taiwan)	730	30 (4.1%)	19 (63.3%)	23 (76.7%)	28 (93.3%)

NA: not available.

∗Based on 90 UC patients meeting the inclusion criteria of pathological samples.

**Table 4:** Detection rates of urothelial carcinomas and upper tract urothelial carcinomas in kidney transplant recipients using urological and imaging methods.

Patients	Test	Positive result (*n*)/total number (*N*)	Detection rate (%)	Reference
Urothelial carcinoma	Urine cytology	2/24	8.3%	Kao et al. [35]
		7/30	23.3%	Wu et al. [40]
		3/11	27.3%	Kliem et al. [53]
		24/21	66.7%	Tsaur et al. [51]
		12/16	75.0%	Liao et al. [37]
		22/27	81.5%	Li et al. [36]
	Cystoscopic biopsy	9/24	37.5%	Kao et al. [35]
	Ureteroscopic biopsy	12/24	50.0%	Kao et al. [35]
	Urine cytology and abdominal ultrasound	4/11	36.3%	Kliem et al. [53]
	Cystoscopy and retrograde pyelography	29/30	96.7%	Wu et al. [40]

Upper tract urothelial carcinoma	Native kidney ultrasound	1/11	9.1%	Li et al. [36]
		1/6	16.7%	Tsaur et al. [51]
		2/14	14.4%	Liao et al. [37]
		2/6	33.3%	Kliem et al. [53]
		15/28	53.6%	Wu et al. [40]
	CT urography	12/14	85.7%	Wang et al. [63]

*n*: number of patients with positive results in each diagnostic test.

*N*: number of patients who underwent each diagnostic test.
